# Biosensing and Delivery of Nucleic Acids Involving Selected Well-Known and Rising Star Functional Nanomaterials

**DOI:** 10.3390/nano9111614

**Published:** 2019-11-14

**Authors:** Susana Campuzano, Maria Gamella, Verónica Serafín, María Pedrero, Paloma Yáñez-Sedeño, José Manuel Pingarrón

**Affiliations:** Departamento de Química Analítica, Facultad de CC. Químicas, Universidad Complutense de Madrid, E-28040 Madrid, Spain; mariagam@quim.ucm.es (M.G.); veronicaserafin@ucm.es (V.S.); mpedrero@quim.ucm.es (M.P.)

**Keywords:** functional nanomaterials, AuNPs, magnetic nanoparticles, janus nanoparticles, AuNWs, nucleic acids, biosensing, delivery

## Abstract

In the last fifteen years, the nucleic acid biosensors and delivery area has seen a breakthrough due to the interrelation between the recognition of nucleic acid’s high specificity, the great sensitivity of electrochemical and optical transduction and the unprecedented opportunities imparted by nanotechnology. Advances in this area have demonstrated that the assembly of nanoscaled materials allows the performance enhancement, particularly in terms of sensitivity and response time, of functional nucleic acids’ biosensing and delivery to a level suitable for the construction of point-of-care diagnostic tools. Consequently, this has propelled detection methods using nanomaterials to the vanguard of the biosensing and delivery research fields. This review overviews the striking advancement in functional nanomaterials’ assisted biosensing and delivery of nucleic acids. We highlight the advantages demonstrated by selected well-known and rising star functional nanomaterials (metallic, magnetic and Janus nanomaterials) focusing on the literature produced in the past five years.

## 1. Introduction

Today, there is an increasing demand for the simple, sensitive, rapid and accurate determination of nucleic acids in fields such as clinical diagnostics, environmental monitoring and food quality control, as well as for the delivery of functional nucleic acids for therapeutic purposes.

Although some of the available conventional methodologies for the determination of nucleic acids provide high sensitivity and sampling frequency, they involve multistep, complex and time-demanding protocols. Within this context, electrochemical and optical nucleic acid biosensing in connection with a wide variety of functional nanomaterials are interesting alternatives for the simple and rapid determination of target nucleic acids meeting the required sensitivity and selectivity in actionable time frames for real-world applications [1,2]. 

It is important to point out that due to its great clinical relevance, the intracellular biosensing of certain nucleic acids, such as miRNAs or mRNA transcripts, is also of particular interest [3]. The currently available intracellular detection methods require long incubation times (~14 h) and a high cell density suspension, which means that thousands of cells are profiled simultaneously, losing important information, including cell identity, and suffering from heterogeneity across cell populations. Accordingly, methods able to perform nucleic acids biosensing at single-cell resolution are urgently needed.

On the other hand, the intracellular delivery of functional nucleic acids, such as RNA transfection agents, able to perform efficient therapeutic actions still needs to overcome important challenges, including the lack of targeting modalities, limited loading efficiency, internalization barriers, and biocompatibility issues [3,4]. In this context, the ultrasound-assisted efficient internalization and intracellular mobility demonstrated by cell viability compatible gold nanowires (AuNWs) proves to be very promising to address such challenges. 

This review critically discusses the capabilities provided by selected well-known and rising star functional nanomaterials for the biosensing and delivery of nucleic acids through a discussion of cutting-edge applications reported mostly in the last 5 years and itemized by the nanoscale material and the type of transduction (optical or electrochemical). This review focuses on these nanomaterials due to the excellent capabilities that gold nanomaterials (nanoparticles, AuNPs, and nanowires, AuNWs) and Janus and/or magnetic type nanodevices exhibit to improve the efficiency and reduce the time of nucleic acid biosensing and delivery through pre-concentration, guided at will, self-propulsion, or cellular internalization, without compromising viability and single-cell resolution. 

## 2. AuNPs, AuNWs, Janus and Magnetic Nanoparticles: Synthesis and Functionalization

AuNPs, AuNWs, Janus and magnetic nanoparticles are functional nanomaterials with very interesting properties to be used in the development of efficient strategies for biosensing and the controlled release of nucleic acids. The main characteristics, opportunities and versatility provided by these four types of nanomaterials in such fields are briefly discussed in the next subsections.

### 2.1. AuNPs

AuNPs, which exhibit outstanding conductivity, chemical inertness and biocompatibility, have been largely studied as electrode materials, catalytic labels, carriers of signal elements and electron transfer regulators to enhance the performance of nucleic acid biosensing [2]. Due to the high applicability of AuNPs, a wide array of solution-based approaches have been developed in the past few decades to control their size, monodispersing, morphology and surface chemistry [5]. However, the protocol most widely employed to prepare dilute solutions of moderately stable spherical AuNPs with diameters between 10 and 20 nm involves treating hydrogen tetrachloroaurate (HAuCl_4_) with citric acid in boiling water, where the citrate acts as both a reducing and stabilizing agent [6].

Considering the large number of reported applications for nucleic acid biosensing using AuNPs and the valuable recent reviews of this topic [2,7], only the most relevant characteristics of AuNPs and a few of the applications considered representative examples of their different uses are discussed in this article.

The use of AuNPs as electrode modifiers provides huge active surface area and facilitates the electron transfer processes at the sensing interface, thereby granting a high sensitivity to biosensors. In addition to the improved conductivity and the catalytic activity of AuNPs, nanostructured surfaces with these nanoparticles greatly enhance the amount of immobilized thiolated probes and allow their favorable orientation and spacing, thus ensuring the target accessibility and improving the hybridization efficiency [2,8,9,10,11]. The great biocompatibility of AuNPs is essential to keep the biological activity of the biomolecules attached to their surface, which produces the attractive storage stability of the prepared bioplatforms [10,11].

AuNPs have also been used as catalytic artificial labels instead of enzymes in nucleic acid biosensing, thus filling critical gaps related to the low thermal and environmental stability of enzymes. Furthermore, unlike the little number of active sites present in enzymes (often just one), AuNPs hold abundant active locus, allowing the production of electrocatalytic signals higher than those obtained with enzymes and therefore, resulting in a higher sensitivity [2,12]. Furthermore, due to AuNPs’ wide specific surface area, they can be filled with a high number of labels and used for amplified detection of nucleic acids. Indeed, AuNPs have been functionalized with unlabeled [13] or redox-labeled [14] nucleic acid probes, redox molecules [15,16], and enzymes or DNAzymes.

The outstanding conductivity of AuNPs has also been used in the development of biosensing methodologies in which the target binding is detected through AuNPs-mediated or inhibited electron transfer [17].

### 2.2. Magnetic Nanomaterials

Both the optical and electrochemical biosensing of nucleic acids in complex matrices require a previous separation of the target nucleotides from their native environment in order to concentrate them and avoid fouling of the transducer surface. The use of magnetic nanomaterials is one of the easiest and most efficient methodologies to perform this step. The magnetic pre-concentration step is also advantageous for eliminating interfering compounds, shortening the analysis time and decreasing stress-induced damages to biomolecules [18]. 

Among the magnetic nanomaterials, superparamagnetic iron oxide nanoparticles (MNPs), with diameters usually ranging between units and various tens of nanometers, have significantly impacted the biosensing of nucleic acids due to the great advantages they provide: large area, easy functionalization and communication with similarly sized biomacromolecules, peroxidase-like activity, rapid assay kinetics, improved sensitivity, matrix effect minimization and easy and effective control of their mobility by an external magnetic field [2,19,20,21,22,23,24,25]. In these biosensing approaches, MNPs can be used as transducer modifiers, as solid nanosupports to perform the affinity assay, which avoids complex and laborious protocols for modifying transducer surfaces [18,26,27,28,29,30], and as labels or nanocarriers of signaling molecules for signal amplification [31,32].

Magnetite (Fe_3_O_4_) and maghemite (γ-Fe_2_O_3_) are the most common MNPs used for biosensing applications [33] due to their biodegradability and high biocompatibility. Diverse physical, chemical, and microbial methods have been proposed for the synthesis of MNPs [34]. Currently, a wide variety of uncoated MNPs or MNPs coated with different functional groups (amine, carboxylic acid, aldehyde, thiol, epoxy, hydroxyl, streptavidin, proteins A and G, albumin, biotin, tosyl) are commercially available. Such coatings minimize agglomeration, confer proper biocompatibility and allow MNPs’ modification with biological molecules such as antibodies, DNA, RNA, and aptamers, which have fueled the development of many biosensing strategies. Regarding core-shell MNPs, Fe_3_O_4_@Au [18,26,27,28,32] and Fe_3_O_4_@SiO_2_ are the most commonly employed. While the presence of Au assures the excellent conductivity and adsorption ability of the nanomaterial and facilitates the functionalization with thiolated molecules through self-assembly [29,35,36], the SiO_2_ surface enhances the stability of nanoparticles and provides a good surface for the binding of bioreagents [37,38]. TiO_2_ and metal-doped iron oxides (MFe_2_O_4_, with M = Co or Mn, among others) have also been explored for biosensing purposes [19,37]. Moreover, coatings of MNPs with polymers such as polyaniline [31] and chitosan [39] allow the increase of the biomolecules’ immobilization capacity. In addition, the combination of MNPs with carbon nanomaterials such as carbon nanotubes or graphene [36] allows the synergetic properties of the resulting nanocomposites to be used profitably. 

### 2.3. Janus Nanoparticles

**Figure 1:**
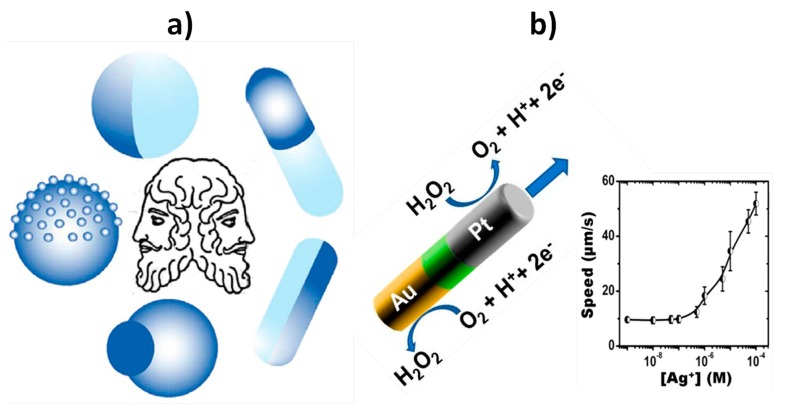
Schematic diagrams illustrating the different morphologies and shapes described for Janus particles (**a**). Au-Pt catalytic nanowires propelled in 5–10% H_2_O_2_ solutions due to a self-electrophoretic mechanism and the dependence of the Au-PtNWs’ speed in 5% H_2_O_2_ solutions containing different concentrations (0.5–100 μM) of AgNO_3_ (b). Reprinted and adapted from [40] (**a**) and [52,54] (**b**), with permission. Copyright RSC, 2016 (**a**), ACS, 2009 and MDPI, 2018 (**b**).

Unlike conventional nanoparticles, two-faced Janus nanodevices, which can be prepared with different morphologies and shapes (Figure 1a) [40], provide anisotropy in structure, constitution, and surface chemistry. These characteristics allow their use to perform complementary functions, achieving outcomes not feasible in the biosensing and cargo delivery areas using conventional nanoparticles [40,41,42,43,44]. Furthermore, analytical functions such as targeting or sensing are spatially decoupled by the surface anisotropy of Janus particles; this allows selective bioconjugation in the spatial environment and provides improved functions and properties such as dual targeting and biosensing [45], not achievable when other single structured or heterogeneous non-Janus nanoparticles are used. This particular performance opens exciting opportunities for the construction of truly multifunctional entities [40,46]. Moreover, the Janus balance, i.e., the surface area ratio derived from the different types of surface compositions in both faces of the nanoparticle, can be modified at will, making these particles a unique category of tailored nanomaterials in contrast to other NPs. There is a wide variety of synthetic strategies available which have evolved rapidly to adapt Janus nanoparticles to different applications. These synthetic methods can be classified as (a) masking or template, (b) direct deposition, (c) phase separation, and (d) self-assembly. Moreover, Janus nanoparticles may incorporate a magnetic material to allow magnetic driving [43,44]. Regarding biosensing, these unique nanoparticles have been exploited as transducer modifiers [47,48] and advanced nanocarriers of signaling molecules for signal amplification [49,50].

One particular type of Janus nanoparticles is Au-Pt catalytic nanowires, prepared by sequential electrodeposition in the membrane templates [51]. These nanowires can self-propel at a speed of around 10 μm s^−1^ in a 5–10% H_2_O_2_ solution. The production of protons and electrons at the extreme of the nanowire due to platinum catalyzed oxidation is responsible for the autonomous nanomotor advancement in the platinum end pointed direction (Figure 1b). It has been shown that the presence of silver ions in the 0.5–100 μM concentration range) provokes a significant speed-up of the nanowires’ movement, this being ascribed to an improvement in the platinum catalytic activity after underpotential silver deposition [52]. Moreover, these nanowires can be made magnetic by incorporating a ferromagnetic nickel segment into the wire (magnetic Janus Au–Ni–Pt nanowires), which makes them easy to guide at will with a simple magnet [53].

### 2.4. AuNWs

AuNWs prepared by a template-directed electrodeposition protocol have demonstrated ultrasound propulsion, rapid penetration through cellular membranes and remain acoustically active in the intracellular space [3,4,55]. The fast cell internalization and rapid intracellular movement under the acoustic field have shown the great potential of AuNWs to perform efficient and accelerated biosensing or delivery tasks inside cells without compromising their viability, even when using a high AuNWs concentration. AuNWs can be employed alone [4] or in combination with other nanomaterials such as graphene oxide [3], and conveniently modified with nucleic acids using thiol chemistry. 

## 3. Biosensing and Delivery of Nucleic Acids: Roles, Opportunities and Cutting-Edge Applications of AuNPs, Magnetic Nanomaterials, Janus Nanoparticles and AuNWs

The unthinkable possibilities offered by the functional nanomaterials commented on in the previous sections have added new dimensions to the efficient biosensing and delivery of functional nucleic acids with cutting-edge applications in connection with optical and electrochemical transduction. As presented in the following subsections, these applications exploit the multiple uses of AuNPs (as transducer modifiers, catalytic labels, nanocarriers and electron transfer regulators), the pre-concentration and on-demand guidance capabilities of magnetic nanomaterials, the dual functionalities of Janus-type nanomaterials and the self-propulsion, internalization and single-cell resolution of AuNWs. 

Table 1 and Table 2 summarize the relevant analytical characteristics of representative cutting-edge applications reported in the last five years for the biosensing and delivery of nucleic acids, exploiting the advantages of functional nanomaterials and using optical and electrochemical detection. The rationale and main features of these applications are critically discussed in the following sections which are classified according to the detection technique and the type/use of the functional nanomaterial.

### 3.1. Optical Biosensing or Delivery of Nucleic Acids Using Functional Nanomaterials

**Figure 2:**
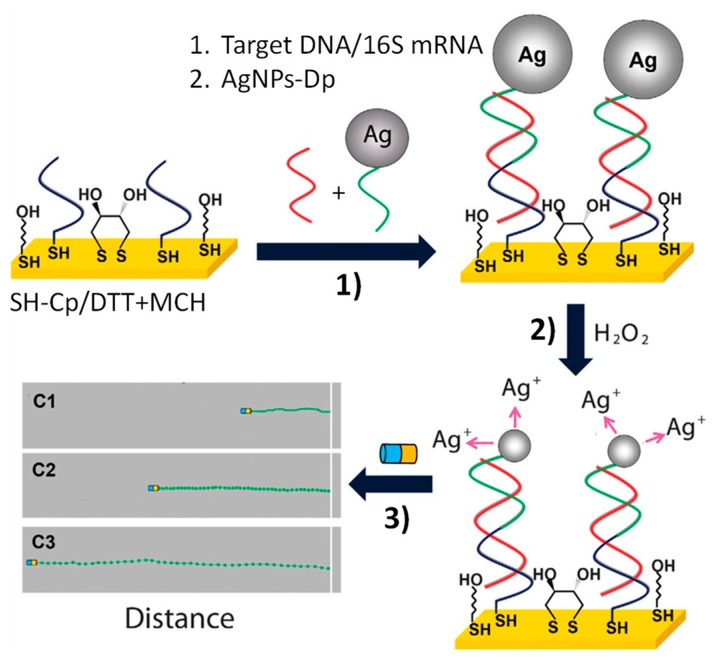
Optical biosensing of nucleic acids using magnetic Janus Au–Ni–Pt nanowires. Sandwich hybridization performed at a 16×AuEs array modified with a ternary self-assembled monolayer (SAM) composed of a thiolated capture probe, dithiothreitol and mercaptohexanol (SH-CP/DTT + MCH) and using a AgNPs-Dp (1). Dissolution of AgNPs tags in the presence of H_2_O_2_, leading to Ag^+^-enriched solution (2). Visual detection of the magnetic Janus Au–Ni–Pt nanowires’ motion in the Ag^+^-enriched solution, resulting after increasing the target nucleic acid concentration (3). Reprinted and adapted from [56] with permission. Copyright Springer Nature, 2010.

Wang’s group (University of California San Diego) was a pioneer in the use of magnetic catalytic Janus Au–Ni–Pt nanowires for the optical biosensing of nucleic acids, profiting from the specific speeding up of these Janus magnetic nanowires when working with Ag^+^ [52]. For the first time, the distance they travelled was used as an analytical signal [56]. The biosensing approach was implemented for the determination of a 30 mer synthetic target DNA or *Escherichia coli* (*E. coli*) 16S mRNA. A sandwich hybridization format was employed on a gold electrode fabricated by photolithography and modified with a ternary monolayer comprising a thiolated specific DNA capture probe, dithiotreitol (DTT) and mercaptohexanol (MCH), and a detector DNA probe modified with Ag nanoparticles (AgNPs). Once the sandwich hybridization assay was completed, a drop of H_2_O_2_ solution was cast on the electrode surface and the AgNPs were rapidly dissolved. The resulting Ag^+^ enriched H_2_O_2_ solution was mixed with an Au–Ni–Pt nanowires suspension and their speed (or the distance travelled) increased with the target nucleic acid concentration and was measured at a defined time (Figure 2). The detection of 40 amol synthetic DNA and 2000 colony forming units (cfu) mL^−1^ of *E. coli* was accomplished directly in raw bacterial lysates, multiple readings being possible in each test, thus reducing the false positives or negatives probability. However, catalytic nanowires’ locomotion is limited to low ionic strength environments, which decreases their utility for biosensing in real media [51,57]. In comparison, acoustically propelled AuNWs, which are cytocompatible, are able to internalize and effectively set in motion in high ionic concentration media and complex biological fluids, providing higher advantages for nucleic acid biosensing [3,58].

The pioneering work of Wang´s group demonstrated the excellent features offered by acoustic propelled AuNWs for single-step biosensing and the delivery of nucleic acids of different natures: microRNAs (miRNAs), messenger RNA (mRNA), silencing RNA (siRNA), and single-guide RNA (sgRNA) at single-cell resolution. 

**Figure 3:**
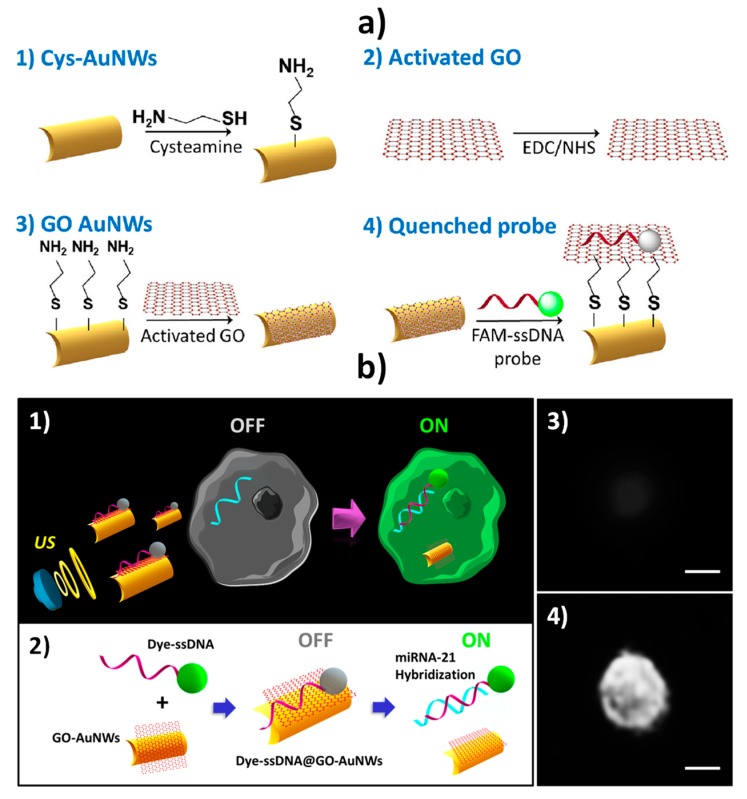
Intracellular biosensing of target miRNA-21 using ultrasound (US)-propelled ssDNA@GO-AuNWs. Schematic illustrations of the steps involved in the ssDNA@GO-AuNWs preparation (**a**) and the “OFF-ON” fluorescent switching system (**b**) for the specific detection of miRNA-21 in intact cancer cells (1); steps involved: immobilization of the dye-ssDNA on the GO-functionalized AuNWs, quenching of the dye fluorescence and fluorescence recovery due to release of the dye-ssDNA from the motor surface upon hybridization with the target miRNA (2); fluorescence images of an MCF-7 cell before (3) and after (4) 20 min incubation with the ssDNA@GO-modified AuNWs under a US field (6 V, 2.66 MHz). Scale bar, 10 μm. Reproduced and adapted from [58] (**a**) and [3] (**b**) with permission. Copyright SAGE Publications, 2019 (**a**) and ACS, 2015 (**b**).

Regarding intracellular miRNA biosensing, graphene oxide (GO)-coated AuNWs (GO-AuNWs), were prepared by covalent immobilization of GO using 1-ethyl-3-(3-dimethylaminopropyl)carbodiimide/N-hydroxysuccinimide (EDC–NHS) chemistry on cysteamine SAM-modified AuNWs. Thereafter, GO-AuNWs were functionalized with a fluorescent dye (fluorescein amidine, 6-FAM)-labeled single-stranded DNA (ssDNA) complementary to the target miRNA (ssDNA@GO-AuNWs) (Figure 3a) [3]. The fluorescence, quenched by π–π interaction between GO and the dye-labeled ssDNA, was recovered upon displacement of the dye-ssDNA probe from the GO-quenching motor surface after cell internalization in the presence of the target miRNA, thus leading to intracellular “OFF–ON” fluorescence switching (see Figure 3b). In a few minutes, the ssDNA@GO-AuNWs discriminated the different expression levels of target miRNA-21 in two types of intact cancer cells (MCF-7 and HeLa). The efficient intracellular propulsion of these functional nanowires accelerated the intracellular hybridization toward rapid “on the move” miRNAs detection with single-cell resolution. Indeed, while ~60% recovery of the fluorescence intensity was observed within 5 min under dynamic conditions, 30 min were required to recover half of the fluorescence under static conditions. The same group recently reported a similar strategy for the detection of human papillomavirus (HPV)–associated oropharyngeal cancer (OPC) by targeting *HPV16 E6* mRNA transcripts [58]. The method allowed the determination of *HPV16 E6* mRNA transcripts both extracellularly (in the total RNA extracted from HPV-positive OPC cells) and within intact OPC cells, with a 2.3 times higher sensitivity working under dynamic conditions compared to static conditions.

**Figure 4:**
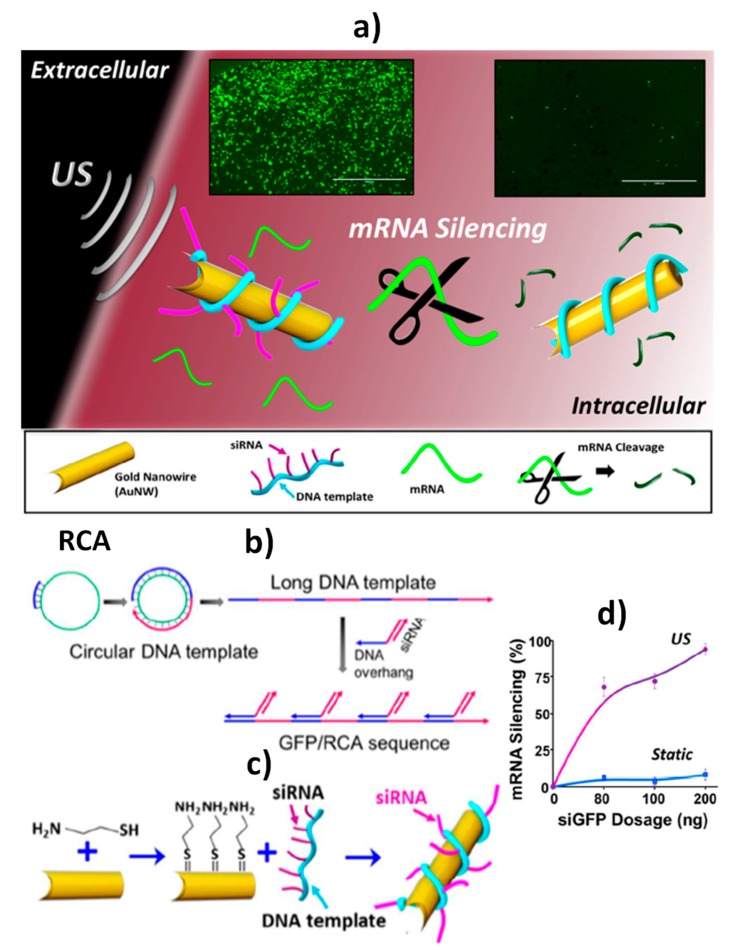
Intracellular delivery of nucleic acids for green fluorescent protein (*GFP*) gene silencing. Schematic diagrams of: GFP/RCA–AuNW penetration inside a cell due to the nanomotor movement under a US field (**a**); rolling circle amplification (RCA) methodology to form, from a short DNA sequence and a circular DNA template, a long ssDNA with repeating units subsequently modified with a complementary DNA overhang-siRNA sequence (**b**); functionalization of the GFP/RCA–AuNWs by cysteamine self-assembly, amine activation and conjugation with GFP/RCA sequence (**c**); dependence of the efficiency of the gene mRNA silencing inside HEK293-GFP cells, using static and US-propelled GFP/RCA–AuNWs, on the siGFP amount immobilized on AuNWs (**d**). Reproduced and adapted from [4] with permission. Copyright ACS, 2016.

Regarding the intracellular delivery of nucleic acids, Wang´s group also pioneered to propose gene silencing strategies effective, rapid and compatible with cell viability, using the knockout of green fluorescent protein (GFP) encoding gene as a model. The strategies involved the use of AuNWs wrapped with a rolling circle amplification (RCA) DNA strand, which served to anchor the siRNA therapy (Figure 4a,b) [4] or loaded with the Cas9–sgRNA complex [59]. The AuNWs were modified with a cysteamine SAM and GFP/RCA (Figure 4c) [4] or with a mercaptopropionic acid (MPA) SAM further incubated with cysteine and EDC–NHS and, thereafter, with the Cas9–sgRNA complex [59]. The use of GFP/RCA-wrapped AuNWs allowed the silencing of 94% of the *GFP* response in two different cell lines (HEK-293 and MCF-7) after 5 min as well as a dramatic (~13-fold) improvement in the silencing response compared to the static modified nanowires (Figure 4d) [4]. Moreover, the Cas9–sgRNA–AuNWs enabled high-efficient knockout with just 0.6 nM of the Cas9–sgRNA complex, displaying more than 80% GFP-knockout within 2 h of cell (B16F10) incubation compared to 30% knockout using their static counterparts (no ultrasound applied). In addition, Cas9–sgRNA–AuNWs exhibited improved cell transfection and faster GFP knockout than that obtained with common lipofectamine-based cell transfection agent [59].

### 3.2. Electrochemical Biosensing of Nucleic Acids Using Functional Nanomaterials.

AuNPs, MNPs and Janus NPs have been widely and successfully exploited in the electrochemical biosensing of nucleic acids. Table 2 summarizes the main analytical characteristics of some highlighted methods discussed below.

A very sensitive PCR-free method for electrochemical biosensing of miRNAs has been proposed using AuNPs as anchoring spots for the ruled and tuned immobilization of the capture probes, improving the biorecognition process efficiency [10]. This method is based on the selective recognition of the RNA/miRNA hybrid formed by the direct hybridization of the target miRNA at an AuNPs-screen-printed carbon electrode (SPCE) modified with a thiolated complementary RNA capture probe and MCH with the viral p19-MBP (maltose binding protein) fusion protein further conjugated with an horseradish peroxidase (HRP)-conjugated anti-MBP antibody. A limit of detection (LOD) of 142 fmol L^−1^ for the synthetic target miRNA was achieved and the method allowed the determination of the endogenous content of miRNA-21 in just 50 ng of the total RNA extracted from cancerous and healthy cells. 

An interesting methodology for the electrochemical detection of *Leishmania* DNA was reported by de la Escosura-Muñiz et al. [12] combining the advantages of magnetic purification/pre-concentration and the use of AuNPs tags for electrochemical detection at the SPCEs. In this method, isothermal amplification of *Leishmania* DNA was performed using primers labeled with magnetic beads (MBs) and AuNPs. The double-labeled amplified products (MB–amplified DNA–AuNP complexes) were magnetically collected on SPCEs and the electrocatalytic activity of the AuNPs tags toward the hydrogen evolution reaction, measured by chronoamperometry, allowed the detection of less than one parasite per microliter of blood.

Attractive strategies for the sensitive electrochemical detection of nucleic acids have also been proposed using AuNPs as nanocarriers of redox-labeled DNA probes [14,15], DNA probes and enzymes [13], and redox reporters [15,16]. Wang et al. [14] reported a sandwich hybridization assay at an Au electrode modified with a thiolated capture probe and the use of AuNPs modified with two types of signaling reporter DNAs labeled with methylene blue (where just one was complementary to the target DNA). The methylene blue signal, measured by differential pulse voltammetry (DPV), enabled the detection of the target DNA in a wide linear range (10^−13^ to 10^−8^ M) with a LOD as low as 50 fM. The use of the non-complementary signaling reporter DNA diluted the reporter probe layer on the AuNPs, thereby reducing the cross-reaction of the functionalized AuNPs and improving the achieved LOD by 20 times. 

**Figure 5:**
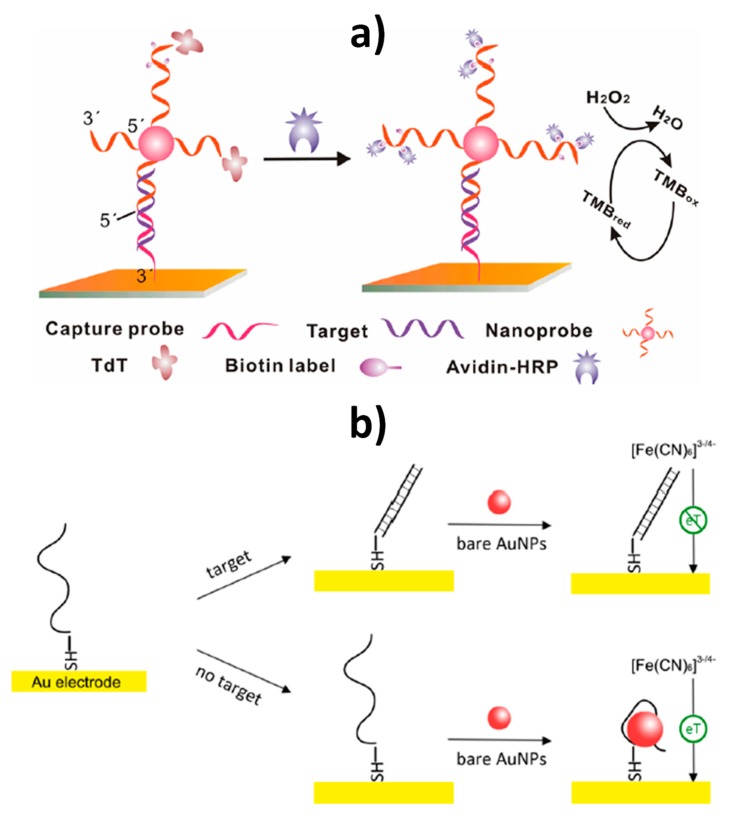
Electrochemical biosensing of nucleic acids based on the use of AuNPs as nanocarriers of probes further elongated with TdT to generate long DNA strands bearing multiple biotin moieties, further conjugated with avidin–HRP complex (**a**), and electron transfer regulators through their non-covalent interaction with ssDNA (**b**). Reprinted from [13] (**a**) and [17] (**b**) with permission. Copyrights ACS, 2015 (**a**) and ACS, 2014 (**b**).

Wan et al. [13] described a detection scheme where the target DNA helped the setup of sandwich complexes between electrode-immobilized capture probes and reporter probe-linked AuNPs. The terminal desoxynucleotidyl transferase (TdT)-catalyzed elongation of the free 3´-terminal of DNA on the AuNPs led to the incorporation of multiple biotin moieties into the generated long DNA strands, which were further used for conjugating avidin-modified HRP molecules (Figure 5a). Using amperometric detection in the presence of 3,3´,5,5´-tetramethylbenzidine (TMB)/H_2_O_2_, the method provided an LOD of 10 fM.

Wang et al. [15] reported a reagent-less strategy for DNA biosensing by exploiting the structure change of electrode-immobilized hairpin DNA probes after hybridization and the subsequent electrochemical signal modification of redox-tagged AuNPs attached to the free end of the hairpin probes. This strategy used AuNPs as nanocarriers with elevated conductivity and surface area for the immobilization of many redox-active melamine–Cu^2+^ complexes, and enabled the DPV detection of the target DNA down to 1.2 × 10^−19^ M. The method was applied to DNA determination in 10% of the spiked human serum samples.

Recently, an enzyme-less sandwich hybridization-based electrochemical biosensor for the determination of miRNAs has been reported [16]. The method made use, for the first time, of SPCEs modified with nanohybrids of rGO with AuNPs as scaffolds to immobilize a thiolated DNA capture probe, as well as of ferrocene (Fc) capped AuNPs modified with streptavidin as non-enzymatic tracers to conjugate the biotinylated DNA detector probe. DPV transduction using Fc as redox reporter was employed. The biosensor exhibited a high sensitivity for the detection of synthetic target miRNA-21 (LOD of 5 fM) and selectivity (single nucleotide discrimination). In addition, it was successfully employed to perform the accurate determination of miRNA-21 in a small amount of cellular RNA_t_ (50 ng) and directly in scarcely diluted serum samples from breast cancer patients.

Yang et al. [17] exploited the use of AuNPs without further functionalization as electron transfer regulators to develop a simple but sensitive electrochemical DNA biosensor. An enhancement of the interfacial electron transfer between the electrode and the redox couple ([Fe(CN)_6_]^3−/4−^) in the solution was observed in the absence of the target DNA, due to the AuNPs serving as conductive bridges over the non-covalent interaction of AuNPs with electrode-immobilized ssDNA probes (Figure 5b). On the contrary, hybridization of the target miRNA with the ssDNA probe hindered the AuNPs–DNA interaction and the consequent enhancement in the electron transfer process. By monitoring changes by electrochemical impedance spectroscopy (EIS), the target DNA, related with the *BRCA1* breast cancer gene, was detected down to 1 pM.

MNPs have been used mainly as nanosupports in the electrochemical biosensing of nucleic acids. Altay et al. [60] reported a direct hybridization method using a DNA capture probe immobilized on amino functionalized carbon coated magnetic nanoparticles (NH_2_-CC-MNPs). The method was applied to determine a synthetic DNA sequence characteristic of the Hepatitis B virus (HBV) by measuring the guanine oxidation signal using DPV and showing a linear range between 5 and 25 μg mL^−1^ and an LOD of 1.15 μg mL^−1^ (20 pmol in 110 μL solution). 

**Figure 6:**
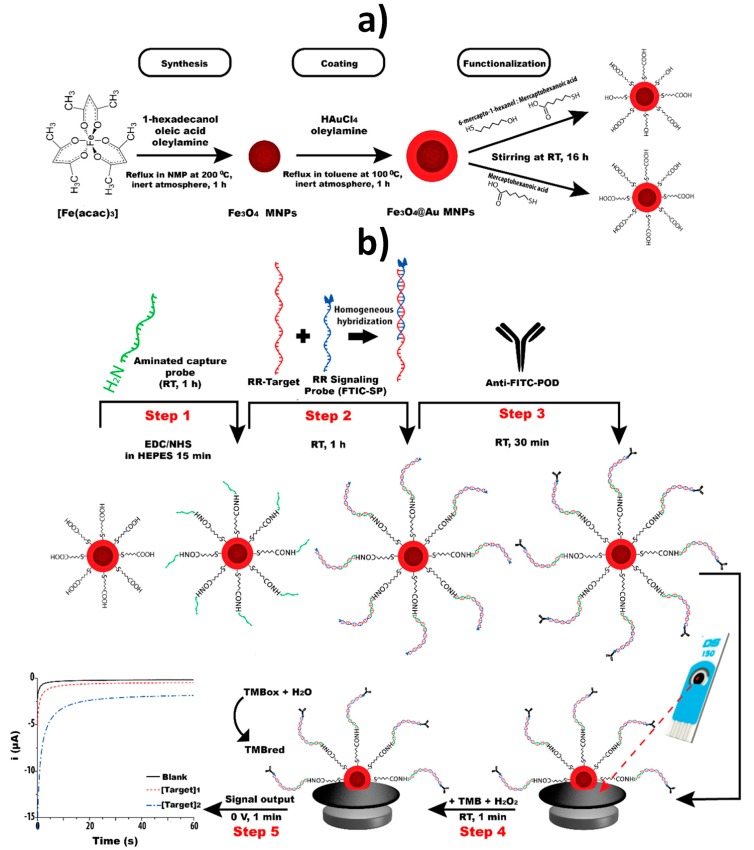
Schematic illustration of an electrochemical biosensing strategy for detecting glyphosate-tolerant soybean, GTS 40-3-2, by implementing a sandwich hybridization format onto Fe_3_O_4_@Au MNPs nanosupports. Synthesis and modification of core-shell Fe_3_O_4_@Au MNPs with a MHA/MCH SAM (**a**). Involved protocol comprised (**b**): covalent immobilization of the NH_2_-Cp to the MHA/MCH-modified-Fe_3_O_4_@Au MNPs through the EDC–NHS reaction (1); homogeneous hybridization between the FITC-modified Dp and the target DNA sequence, and subsequent heterogeneous hybridization reaction with NH_2_-Cp-MHA/MCH-modified-Fe_3_O_4_@Au MNPs (2); enzymatic labelling with HRP-antiFITC Fab fragments (3); and chronoamperometric detection using the TMB–H_2_O_2_ system at SPCEs upon magnetic capture of the modified MNPs on the working electrode (4) and (5). Reprinted from [29] with permission. Copyright Elsevier, 2018.

Fe_3_O_4_@Au MNPs have been used as nanosupports for the implementation of sandwich-type hybridization assays for the detection of genetically modified organisms (GMOs). Fe_3_O_4_@Au MNPs were synthesized using a two-step procedure consisting of the Fe_3_O_4_ MNPs synthesis by thermal decomposition, followed by coating with a gold shell via chemical reduction of Au (III) in the presence of a reducing capping agent (oleylamine). The methods involved covalent immobilization of DNA capture probes modified with amino groups (NH_2_-Cps) through EDC–NHS chemistry on the surface of Fe_3_O_4_@Au MNPs modified with SAMs prepared from different mercaptoacids such as thioctic acid (TOA) [18] or 6-mercaptohexanoic acid (MHA) [29,30,61] with [18,30,61] or without [29] post-treatment with MCH (Figure 6a). The detector probes (Dps) were tagged with fluorescein isothiocyanate (FITC) and digoxigenin (DIG) and then with Fab fragments of antibodies specific to those tags conjugated to HRP. In all cases, the detection was performed by chronoamperometry using the TMB–H_2_O_2_ system after the magnetic capture of the modified MNPs on the surface of commercial SPCEs [18,29,30] or homemade gold [61] electrodes (Figure 6b). These methods provide LODs in the pM–nM range for the synthetic DNA sequence, reliability for single [18,29,61] or dual [30] determination, and were applied to the detection of genetic modified soybean and maize events in PCR amplicons from certified reference materials [18], in cat feed, soybean seeds and maize flour samples [29,61]. It is also worth remarking that while the assay time starting from Cp-Fe_3_O_4_@Au MNPs is ~2 h, the synthesis of the Fe_3_O_4_@Au MNPs requires ~20 h and their modification with the SAM and Cp ~24 and 2 h, respectively. 

**Figure 7:**
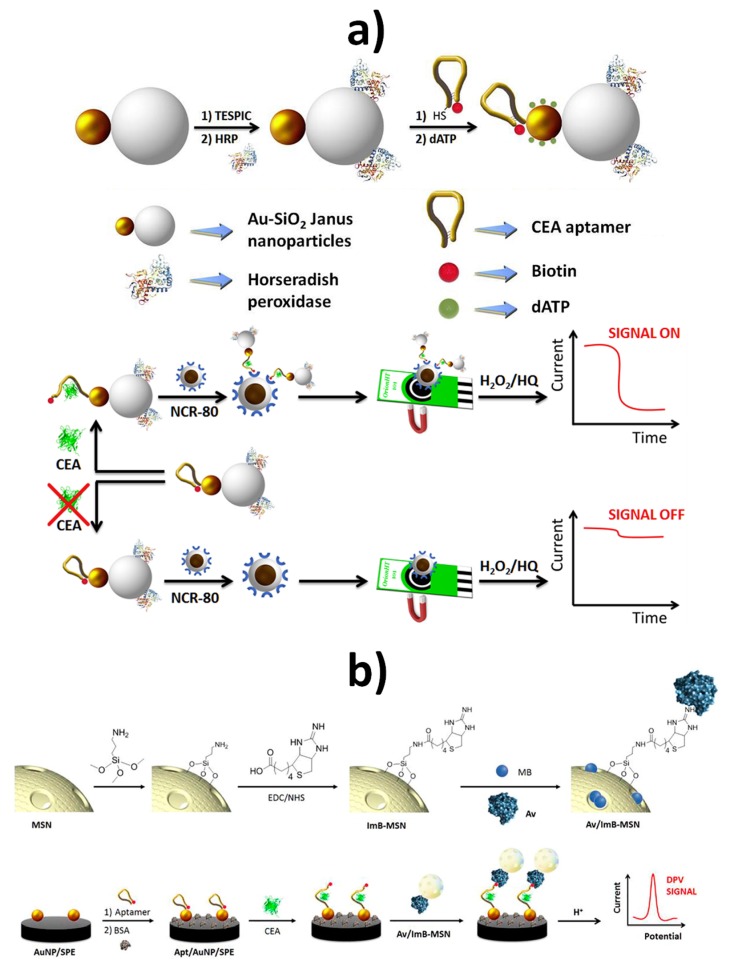
Electrochemical aptasensing of CEA involving the use of Janus Au-mesoporous silica nanoparticles (**a**) and mesoporous silica nanoparticles (**b**) as nanolabels or nanocarriers of signaling elements, respectively. Schematic display of the protocols used to functionalize the nanoparticles (top) and their use for electrochemical CEA aptasensing (down). Reprinted and adapted from [49] (**a**) and [50] (**b**) with permission. Copyrights Elsevier, 2019 (**a**,**b**).

Janus Au-mesoporous silica nanoparticles (Au-MS JNPs) were previously exploited as scaffolds to design integrated electrochemical enzymatic biosensors [47,48]. Recently, they have also been used as labels for signal amplification in the electrochemical aptasensing of carcinoembryonic antigen (CEA) [49]. The strategy involved proper functionalization of Janus nanoparticles silica surfaces with HRP to act as signaling element while the Au face was modified with a dual biotin thiol-functionalized anti-CEA DNA hairpin aptamer (Figure 7a top). The specific recognition of CEA by the bi-functionalized Janus nanoparticles provoked unfolding of the hairpin aptamer, thus unmasking the biotin residues and allowing the recognition of the CEA–Janus nanoparticle complex by avidin-modified Fe_3_O_4_@SiO_2_ NanoCaptors^®^ (NCR-80). This resulted in an enhanced amperometric signal in the presence of H_2_O_2_/HQ (hydroquinone) upon their magnetic capture on the working electrode of SPCEs (Figure 7a down). This aptasensor was able to detect CEA in the range from 5.5 pM to 28 nM, with a LOD of 1.2 pM and was applied to the analysis of spiked lyophilized human serum [49]. A closely related method has been proposed recently by the same group using mesoporous silica nanoparticles (MSNs) loaded with the redox probe methylene blue and capped with an avidin–imminobiotin (Av–ImB) stimulus–responsive gate-like ensemble (Figure 7b top) as the signal amplification element in connection with an aptasensor for CEA constructed by attaching a biotin and thiol-functionalized anti-CEA DNA hairpin aptamer on AuNPs–SPCEs [50]. In this case, the unfolding of the aptamer molecule attached to the AuNPs–SPCE after CEA specific recognition leaves biotin residues free to further associate with the avidin-capped mesoporous nanocarrier (Figure 7b down). By DPV measurement of the acid-assisted released encapsulated methylene blue, CEA could be determined over the 1.0 pg mL^−1^ to 160 ng mL^−1^ range in five-fold diluted human serum samples.

## 4. Main Conclusions, Challenges and Perspectives

The current generation of functional nanomaterials (AuNPs, magnetic nanomaterials, Janus nanoparticles and AuNWs) allows efficient and rapid static and dynamic biosensing and delivery of nucleic acids at both extracellular and intracellular levels. The unique superparamagnetic anisotropy and self-propulsion properties demonstrated by the selected nanomaterials, mostly metallic, as compared with other carbon and/or polymeric counterparts, are decisive in meeting these particularly challenging applications.

The self-propulsion of metallic NWs (Au-Pt and Au) has brought a new paradigm in bioanalysis, proposing, for the first time, the use of the speed/distance of the catalytic Janus Au-Pt ones as analytical signals and the possibility to perform biosensing or delivery in significantly shorter times (just a few minutes compared to hours), allowing the development of near real-time biosensing strategies.

The unique properties and versatility of the use of AuNPs has made it possible to exploit them as electrode materials, catalytic labels, carriers of signal elements or electron transfer regulators in a variety of biosensing methods for the determination of nucleic acids of different natures (mostly DNAs and miRNAs). Their use as electrode materials exploits their unique features to facilitate electronic transfer processes on the electrode surface and provide a large active surface for the immobilization of a large number of thiolated probes with adequate orientation and spacing, thus ensuring maximum efficiency in the hybridization reaction. Also, their great biocompatibility, essential to keep the biological activity of biomolecules attached to their surface, imparts remarkable storage stability to the developed bioplatforms.

In recent years, the superparamagnetic properties of MNPs and, particularly of Fe_3_O_4_@Au MNPs, have been exploited for their use as efficient nanosupports in the development of nucleic acids biosensing strategies with improved sensibility, reduced test time and applicability in complex matrices after minimal test pretreatments. On the other hand, the spatial-selective functionalization of Janus Au-mesoporous silica nanoparticles has been utilized recently to propose attractive nanocarriers of signaling elements in the aptasensing of cancer biomarkers.

Acoustically powered AuNWs have been demonstrated to be active vehicles for efficient cytosolic delivery of active nucleic acids in comparison with the common diffusion-passive methods, thus offering an attractive way to enhance the current landscape of intracellular payloads delivery (at genetic, regulatory and functional levels) for therapeutic purposes and to perform efficient and near real-time biosensing, both extracellularly and intracellularly, without damaging the cells. Although still described to-date as proof-of concept studies, the use of AuNWs as cargo transport vehicles to the cells inside has been demonstrated to overcome physiological barriers for the intracellular delivery of functional molecules (nucleic acids, proteins or sperm cells), thus paving the way for developing highly efficient therapeutic or fertilization applications. 

Despite the great advances that have been witnessed in recent years, in order to exploit even further the great potential of these nanoscaled materials and the differential properties they offer with respect to other types of carbon- or polymer-based nanomaterials, additional efforts should be focused on i) maximum simplification of the protocols involved to move from proof-of-concept to more real applications and from laboratory-based to point-of-care test settings, ii) facilitate targeted delivery to specific locations by the incorporation of specific receptors on the nanomaterials surface, and iii) developing methods for multiplexing or co-delivering of multiple payloads. Furthermore, bearing in mind that the manufacturing and/or modification processes of these functional nanomaterials are fairly long (between 16 and 75 h from information given in Table 1 and Table 2), exhaustive studies should be carried out to evaluate their operational stability and variability among different batches prepared in the same way. The improved efficiency and speed for the biosensing or delivering of nucleic acids provided by functional nanomaterials also allows envisioning the development of naked-eye approaches without the need for complex instrumentation. 

In recent years, there has been a dizzying progress in the flourishing of new functional nanomaterials, the number of methods available for their preparation and modification, and the combination of different nanomaterials to exploit synergistic properties. These great developments, along with the versatility of use they have as surface modifiers, nanosupports and nanolabels/nanocarriers allow the foresight of a high number of innovative designs/combinations of functional nanomaterials and unlimited opportunities, avenues and applications to overcome current and unexpected issues in the highly relevant processes involving biosensing or controlled transport of nucleic acids in real-world applications.

## Figures and Tables

**Table 1:** Optical biosensing or delivery of nucleic acids using functional nanomaterials.

Objective	Functional Nanomaterial	Rationale Behind the Strategy	Detection Technique	Analyte Detected/Delivered	LOD	Sample	Assay Time, min	Ref.
Biosensing	Magnetic Janus Au–Ni–PtNWs	Increased speed of Au–Ni–PtNWs in the presence of Ag^+^ enriched H_2_O_2_ solution generated by performing sandwich hybridization assays at photolithography-prepared 16×AuEs array involving an AgNPs-labeled detector probe (AgNPs-Dp)	Optical	DNA and *E. coli* 16S mRNA	40 amol synthetic DNA and 2000 cfu·mL^−1^ of *E. coli*	Raw bacterial lysate	~60 min starting from Cp-16×AuEs array (preparation of Au–Ni–Pt NWs: ~2 h; AgNPs-Dp: ~62 h; Cp-16×AuEs array: ~13 h)	[56]
Biosensing (in vitro and intracellular)	GO-AuNWs	“Off–On” fluorescence switching due to the displacement of the dye-ssDNA probe from the ssDNA@GO-AuNWs surface after cell internalization in the presence of the target miRNA	Fluorescent	miRNA-21	Single cell	Intact cancer cells (MCF-7 and HeLa)	5–10 min starting from ssDNA@GO-AuNWs (AuNWs: ~2 h; ssDNA@GO-AuNWs ~14 h 15 min)	[3]
Biosensing (in vitro and intracellular)	GO-AuNWs	“OFF–ON” fluorescence switching due to the displacement of the dye-ssDNA probe from the ssDNA@GO-AuNWs surface after cell internalization in the presence of the target mRNA	Fluorescent	HPV16 E6 mRNA transcripts	Single cell	Total RNA extracted from HPV-positive OPC cells and intact cells (HPV-positive or HPV-negative human OPC cells)	15 min starting from ssDNA@GO-AuNWs (AuNWs: ~2 h; ssDNA@GO-AuNWs ~14 h 15 min)	[58]
Delivery	siRNA/RCA- AuNWs	Intracellular delivery of GFP/RCA to knockout *GFP* gene	Fluorescent	siRNA/RCA	Single cell	Intact HEK-293 and MCF-7 cells	~5 min starting from siRNA/RCA- AuNWs (AuNWs: ~2 h; RCA: ~19 h 45 min; siRNA/RCA- AuNWs: ~13 h)	[4]
Delivery	Cas9–sgRNA–AuNWs	Intracellular delivery of Cas9–sgRNA complex to silence the *GFP* response	Fluorescent	Cas9–sgRNA complex	Single cell	Intact B16F10 cells	~5 min starting from Cas9–sgRNA–AuNWs (AuNWs: ~2 h; Cas9–sgRNA complex: ~10 min; Cas9–sgRNA–AuNWs: ~16 h)	[59]

cfu: colony forming units; GFP: green fluorescent protein; GO: graphene oxide; HPV: human papillomavirus; OPC: associated oropharyngeal cancer; LOD: limit of detection; RCA: rolling circle amplification.

**Table 2:** Electrochemical biosensing of nucleic acids using functional nanomaterials

Electrode	Functional Nanomaterial (Role)	Method	Detection Technique	Target Analyte	Linear Range	LOD	Sample	Assay Time, Min	Ref.
AuNPs–SPCE	AuNPs (electrode modifier)	Direct hybridization approach at SH-RNA-Cp/MCH-AuNPs–SPCE and selective recognition of the RNA/miRNA hybrid with the p19-MBP fusion protein further conjugated with and HRP anti-MBP antibody	Amperometry (H_2_O_2_/HQ)	miRNA-21	0.5–50 pmol·L^−1^	142 fmol·L^−1^	RNA_t_ extracted from healthy and cancerous breast cells	~60 min starting from SH-RNA-Cp/MCH-AuNPs–SPCE (SPCE modification: ~9 h 5 min)	[10]
SPCE	AuNPs (catalytic label)	Isothermal amplification of *Leishmania* DNA using primers labeled with MBs and AuNPs and magnetic capture of the MB–amplified DNA–AuNP complexes on SPCEs	Chronoamperometry	*Leishmania* DNA	500–0.5 parasite mL^−1^ blood	0.8 parasite mL^−1^ blood	Dog’s blood	~10 min starting from primers conjugated with MBs and AuNPs (primers conjugation: ~65 h 55 min)	[12]
AuE	AuNPs (nanocarriers of redox-labeled DNA probes)	Sandwich hybridization assay developed at an Au electrode modified with thiolated Cps; use of AuNPs modified with two different probes labeled with methylene blue (just one complementary to the target DNA)	DPV (methylene blue)	Target DNA	10^−13^–10^−8^ M	50 fM	—	~2 h starting from SH-Cp/MCH-AuE (modified AuE: ~1 h and DNA-AuNPs conjugates: ~5 h 30 min)	[14]
AuE	AuNPs (nanocarriers of reporter probes and enzymes)	Sandwich hybridization between SH-Cp/SH-OEG-AuE and reporter probe-linked AuNPs, and terminal deoxynucleotidyl transferase (TdT)-catalyzed elongation of the free 3´-terminal of DNA on the nanoprobe to incorporate multiple biotin moieties further conjugated with avidin-modified HRP molecules	Amperometry (TMB/H_2_O_2_)	Target DNA	10 fM–10 pM	10 fM	—	~2 h 45 min starting from SH-Cp/SH-OEG-AuE (modified AuE: ~16 h and DNA-AuNPs conjugates: ~56 h 15 min)	[13]
AuE	AuNPs (nanocarriers of melamine–Cu^2+^ complexes)	Hybridization-induced structural variation of electrode-immobilized SH-hCp with attached Cu^2+^-Mel-AuNPs	DPV (Cu^2+^/Cu^+^)	Target DNA	1.0 × 10^−18^ M–1.0 × 10^−12^ M	1.2 × 10^−19^ M	10% spiked human serum	~40 min starting from Cu^2+^-Mel-AuNPs/SH-hCp/MCH/AuE (AuE modification: ~77 h 20 min)	[15]
AuNPs/rGO/SPCEs	AuNPs (nanocarriers of Strep and Fc)	Sandwich hybridization approach at a MCH/HS-DNACp-AuNPs/rGO/SPCEs using a biotinylated Dp conjugated with Fc-AuNPs-Strep conjugates	DPV (Fc)	miRNA-21	10 fM–2 pM	5 fM	RNA_t_, extracted from breast adenocarcinoma cells and serum from cancer patients	~1 h 45 min starting from Fc-AuNPs-Strep (AuNPs modification: ~24 h and HS-DNACp-AuNPs/rGO/SPCE: ~9 h 30 min)	[16]
AuE	AuNPs (electron transfer regulator)	Enhancement of the interfacial electron transfer process between the electrode and the redox couple ([Fe(CN)_6_]^3−/4−^) in the absence of target DNA due to AuNPs–DNA binding	EIS ([Fe(CN)_6_]^3−/4−^)	Target DNA (*BRCA1* gene)	1 pM–500 nM	1 pM	—	~2 h starting from AuNPs (AuNPs preparation: ~30 min and HS-DNACp-AuE: ~3 h)	[17]
PGE	NH_2_-CC-MNPs	Direct DNA hybridization at DNA Cp immobilized onto NH_2_-CC-MNPs	DPV (guanine oxidation)	HBV target DNA	5–25 μg mL^−1^	1.15 μg mL^−1^	—	~35 min starting from Cp-NH_2_-CC-MNPs (synthesis: ~23 h 30 min + Cp immobilization: ~1 h 20 min)	[60]
SPCE	Fe_3_O_4_@Au MNPs	Sandwich hybridization approach involving covalent immobilization of an NH_2_-DNA Cp onto Fe_3_O_4_@Au MNPs modified with a TOA/MCH SAM and a FITC signaling probe further conjugated with anti-FITC-HRP Fab fragment	Chronoamperometry (TMB/H_2_O_2_)	GMO (a specific fragment of the transgenic construct from maize MON810 maize)	0.25–2.5 nM	0.15 nM	PCR amplicons obtained from CRMs of maize MON810	~2 h starting from Cp-Fe_3_O_4_@Au MNPs (MNPs synthesis: ~20 h;TOA/MCH SAM: ~24 h; Cp immobilization: ~2 h)	[18]
SPCE	Fe_3_O_4_@Au MNPs	Sandwich hybridization approach involving covalent immobilization of an NH_2_-DNA Cp onto Fe_3_O_4_@Au MNPs modified with a MHA/MCH SAM and a FITC signaling probe further conjugated with anti-FITC-HRP Fab fragment	Chronoamperometry (TMB/H_2_O_2_)	DNA fragments from the insertion point of the transgenic construct of RR GTS 40-3-2 soybean, an event-specific sequence, and of the taxon-specific soybean gene, lectin	0.1–10.0 nM (event specific) 0.1–5.0 nM (taxon-specific)	0.02 nM (event specific) 0.05 nM (taxon-specific)	PCR amplicons obtained from soybean seeds and cat feed	~1 h 40 min starting from Cp- Fe_3_O_4_@Au MNPs (synthesis: ~21 h; MHA/MCH SAM: ~16 h; Cp immobilization: ~1 h 40 min)	[29]
SPdCE	Fe_3_O_4_@Au MNPs	Sandwich hybridization approaches involving covalent immobilization of NH_2_-DNA capture probes onto Fe_3_O_4_@Au MNPs modified with a MHA SAM and FITC or DIG signaling probes further conjugated with anti-FITC-HRP or anti-DIG-HRP Fab fragments	Chronoamperometry (TMB/H_2_O_2_)	GMO (fragments of the transgenic construct from GTS 40-3-2 and MON89788 soybean lines)	0.1–2.5 nM (GTS 40-3-2) 0.1–1.0 nM (MON89788)	0.1 nM (both events)	—	~2 h 5 min starting from Cp- Fe_3_O_4_@Au MNPs (synthesis: ~21 h; MHA SAM: ~16 h; Cp immobilization: ~1 h 35 min)	[30]
Homemade AuE	Fe_3_O_4_@Au MNPs	Sandwich hybridization approach involving covalent immobilization of an NH_2_-DNA Cp onto Fe_3_O_4_@Au MNPs modified with a MHA/MCH SAM and a FITC signaling probe further conjugated with anti-FITC-HRP Fab fragment	Chronoamperometry (TMB–H_2_O_2_)	Maize taxon-specific (HMGA gene)	0.5–5 nM	90 pM	PCR amplicons obtained from maize flour	~2 h starting from Cp-Fe_3_O_4_@Au MNPs (synthesis: ~20 h; MHA/MCH SAM: ~24 h; Cp immobilization: ~2 h)	[61]
SPCE	Au-MSN JNPs	Au-MS JNPs functionalized with HRP and a dual biotin thiol-functionalized anti-CEA DNA hairpin aptamer in connection with avidin-modified Fe_3_O_4_@SiO_2_ NanoCaptors	Amperometry (H_2_O_2_/HQ)	CEA	5.5 pM–28 nM	1.2 pM	Spiked lyophilized human serum samples	~1 h starting from bifunctionalized Au-MSN JNPs (synthesis of Au-MSN JNPs: ~38 h 30 min; bifunctionalization: ~4 h 5 min)	[49]
AuNPs–SPCE	MSNs	MSNs loaded with methylene blue molecules and capped with an avidin/imminobiotin stimulus-responsive gate-like ensemble in connection with an AuNPs–SPCE modified with a biotin and thiol-functionalized anti-CEA DNA hairpin (Apt-AuNPs–SPCE)	DPV (methylene blue)	CEA	1.0 pg mL^−1^–160 ng mL^−1^	280 fg mL^−1^	5-fold diluted human serum samples	~45 min starting from bifunctionalized MSNs and Apt-AuNPs–SPCE (MSNs: ~2 h 5 min; bifunctionalization: ~72 h; Apt-AuNPs–SPCE: ~1 h 5 min)	[50]

Au-MS JNPs: Janus Au-mesoporous silica nanoparticles; CEA: carcinoembryonic antigen; Cp: capture probe; CRMs: certified reference materials; DIG: digoxigenin; Dp: detector probe; DPV: differential pulse voltammetry; EIS: electrochemical impedance spectroscopy; Fc-AuNPs-Strep: Ferrocene capped gold nanoparticle-streptavidin conjugates; FITC: fluorescein isothiocyanate; GMO: genetic modified organism; hCp: hairpin capture probe; HER: hydrogen evolution reaction; HBV: Hepatitis B virus; HQ: hydroquinone; HRP: horseradish peroxidase; Mel: melamine; MBs: magnetic beads; MBP: maltose binding protein; MCH: mercaptohexanol; MHA: 6-mercaptohexanoic acid; MSNs: Mesoporous silica nanoparticles; NH_2_-CC-MNPs: amino functionalized carbon coated magnetic nanoparticles; OEG: oligo(ethylene)glycol; PGE: pencil graphite electrode; SAM: self-assembled monolayer; RR: Roundup Ready; SPCE: screen-printed carbon electrode; SPdCE: screen-printed dual carbon electrodes; Strep: streptavidin; TMB: 3,3′,5,5′-Tetramethylbenzidine; TOA: thioctic acid.
